# The Challenges and Opportunities of Protein Coronas for Nanoscale Biomolecular Sensing

**DOI:** 10.1002/smll.202503820

**Published:** 2025-07-26

**Authors:** Samuel Cheeseman, Parisa Moazzam, Negar Mahmoudi, Morteza Mahmoudi, Frank Caruso, Antonio Tricoli, David R. Nisbet

**Affiliations:** ^1^ The Graeme Clark Institute The University of Melbourne Parkville Victoria 3010 Australia; ^2^ Department of Biomedical Engineering Faculty of Engineering and Information Technology The University of Melbourne Parkville Victoria 3010 Australia; ^3^ School of Science STEM College RMIT University Melbourne Victoria 3001 Australia; ^4^ Nanotechnology Research Laboratory Faculty of Engineering University of Sydney Sydney New South Wales 2006 Australia; ^5^ Precision Health Program Michigan State University East Lansing MI 48824 USA; ^6^ Depatment of Radiology College of Human Medicine Michigan State University East Lansing MI 48824 USA; ^7^ Department of Chemical Engineering The University of Melbourne Parkville Victoria 3010 Australia

**Keywords:** biofluids, biomolecular sensors, fouling, nanoscale, personalized corona, protein corona

## Abstract

The unique benefits of sub‐picomolar and single‐molecule detection for the diagnosis and prognosis of diseases and therapeutic efficacy monitoring have been driving the development of nanoscale biomolecular sensors. Nanoscale sensors can be attached to the surface or dispersed in solution, enabling the rapid detection of analytes with high sensitivity and specificity by overcoming concentration‐driven diffusion limits. In biological fluids, however, nanoscale objects are surrounded by biomolecules, mostly proteins, that form an evolving encapsulating surface layer, commonly known as the protein corona. The protein corona can modify the biosensor surface, which can adversely impact biosensing specificity, sensitivity, and accuracy. Conversely, the protein corona can be exploited to design biosensors for disease diagnostics, the discovery of new biomarkers, and environmental contaminants. In this review, the factors influencing protein corona formation on nanoscale biosensors are examined. Characterization methods and the effects of protein corona formation on the performance of nanoscale biosensors are also discussed. Promising strategies to prevent, circumvent, and exploit corona formation are presented and this review concludes by outlining future perspectives of nanoscale biomolecular sensors for practical application in biological fluids.

## Graphical abstract

Nanoscale biomolecular sensors, or “nanobiosensors”, have enabled rapid detection of analytes with high sensitivity and specificity. In biological fluids, however, nanoscale objects are surrounded by biomolecules, that form an evolving encapsulating surface layer, commonly known as the protein corona. In this review, the challenges, opportunities, principles, and analysis techniques associated with protein corona formation on nanobiosensors are critically examined.

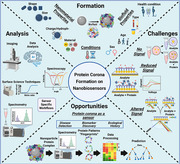

## 1 Introduction

The rapid advancement of nanotechnology over the early 21st century has driven the development of nanoscale biomolecular sensors that enable the detection of analytes with high sensitivity, specificity, and speed.^[^ ^1^, ^2^, ^3^ ^]^ To be effective, these nanobiosensors need to operate in complex biological fluids. The high concentration of biomolecules (particularly proteins) in solution can adsorb onto the surface of nanomaterials, forming a dynamic encapsulating layer commonly referred to as the protein corona.^[^ ^4^, ^5^, ^6^ ^]^ This layer can affect the performance of nanobiosensors, whilst also creating a new class of sensor specifically exploiting this phenomenon. While there are many reviews focusing on the formation and effects of the protein corona on many aspects of nanomedicine, such as drug delivery, a review summarizing the ramifications of the protein corona on nanobiosensors is lacking.

Biosensors consist of a biological recognition element integrated with a transducer.^[^ ^7^ ^]^ A well‐known example of a biosensor includes glucose sensors,^[^ ^8^ ^]^ where glucose oxidase acts as the biomolecule recognition element, and a working electrode transduces this recognition into an electrical signal.^[^ ^9^ ^]^ Some applications require more selectivity, sensitivity, and/or faster response than traditional biosensors can offer, necessitating technological advancement.^[^ ^10^, ^11^ ^]^ In these cases, current technologies encounter a fundamental limit based on the concentration‐limited diffusion flux of the targeted biomolecule toward a surface.^[^ ^12^ ^]^ One promising strategy to overcome the diffusion limit is through the development of nanobiosensors.^[^ ^13^ ^]^ Several terms have been used in the literature to define these types of sensors, such as nanosensors, nanomaterials, and nanoparticle (NP) biosensors. In this review, we define nanobiosensors as a device that incorporates a component that interacts with the tested fluid and a transduction mechanism, featuring at least one dimension in the nanoscale. The use of nanomaterials increases the relative surface area for interaction with the target analyte and can have other advantages such as operating as both the recognition and transduction elements^[^ ^1^, ^12^ ^]^ and shortening the diffusion path of the target analyte to the sensor surface.^[^ ^13^ ^]^

For nanobiosensors to be effective they need to operate in complex biological fluids such as plasma, serum, blood, saliva, liquid biopsies, urine, and cell lysate.^[^ ^14^, ^15^, ^16^ ^]^ However, proteins and other biomolecules present in these biological fluids can bind to the surface of nanoscale objects, resulting in the formation of a corona.^[^ ^4^, ^5^, ^6^ ^]^ A large range of biomolecules can adsorb onto NP surfaces, with the predominant species being proteins.^[^ ^6^, ^17^ ^]^ This corona fouls NPs and can modify their properties such as size, charge, and wettability, thereby influencing the performance of nanobiosensors. This review will refer to this phenomenon as the “protein corona” as NP interactions with proteins represent the majority of current research. In a recent perspective, Li et al., highlight progress made in the biomolecule corona field, the complexities of navigating the biomolecule corona, and opportunities to improving our understanding of the biomolecule corona to guide nanomedicine design.^[^ ^17^ ^]^

Here, we review the literature that explores the influence of the protein corona on nanobiosensor performance in complex biological fluids and aims to provide a useful overview of the research to date. First, we summarize the need for nanobiosensors, then provide a detailed overview of the factors influencing protein corona formation on nanobiosensors and discuss the challenges arising from this. We then present the key characterization techniques and strategies to mitigate the adverse effects of protein corona formation on nanobiosensors. Finally, we discuss emerging strategies for exploiting NP corona formation for nanobiosensors, challenges, and future perspectives.

## 2 The Need for Nanobiosensors

The development of point‐of‐care diagnostics for the detection of very low‐concentration biomarkers in complex biological fluids is becoming increasingly important for disease diagnostics, prognostics, and tracking treatment efficacy. This detection is made more difficult by the high dynamic range of analytes in biological fluids. For example the protein concentration in blood plasma can vary by ≈16 orders of magnitude^[^ ^18^, ^19^ ^]^ where important biomarkers such as cytokines (e.g. interleukins and interferons), can be present in the blood plasma at concentrations at or below pg mL^−1^,^[^ ^20^ ^]^ while human serum albumin is typically present in the mg ml^−1^ range. Another example is liquid biopsies, which involve the detection of cancer biomarkers such as RNA, DNA, circulating tumor cells (CTCs), lipids, metabolites, proteins, cytokines, exosomes, and T cells found in very low concentrations in the blood, which provide information on tumors.^[^ ^21^ ^]^ These markers require sub‐picomolar detection limits among an abundance of healthy cells and non‐target biomolecules that are present in blood.^[^ ^11^, ^22^ ^]^ Liquid biopsies have been shown as a useful tool in a range of cancers including gastric,^[^ ^23^ ^]^ melanoma,^[^ ^24^ ^]^ breast,^[^ ^25^ ^]^ colorectal, and lung,^[^ ^26^ ^]^ where compared to solid biopsies, they provide a less invasive method for diagnosing diseases, monitoring tumor progression, and understanding resistance to treatments and medication toxicity.^[^ ^27^ ^]^ Finally, some bloodborne bacterial infections require detection of <10 units per milliliter against a background of nonspecific biomolecules and healthy cells.^[^ ^11^ ^]^

**Figure 1:**
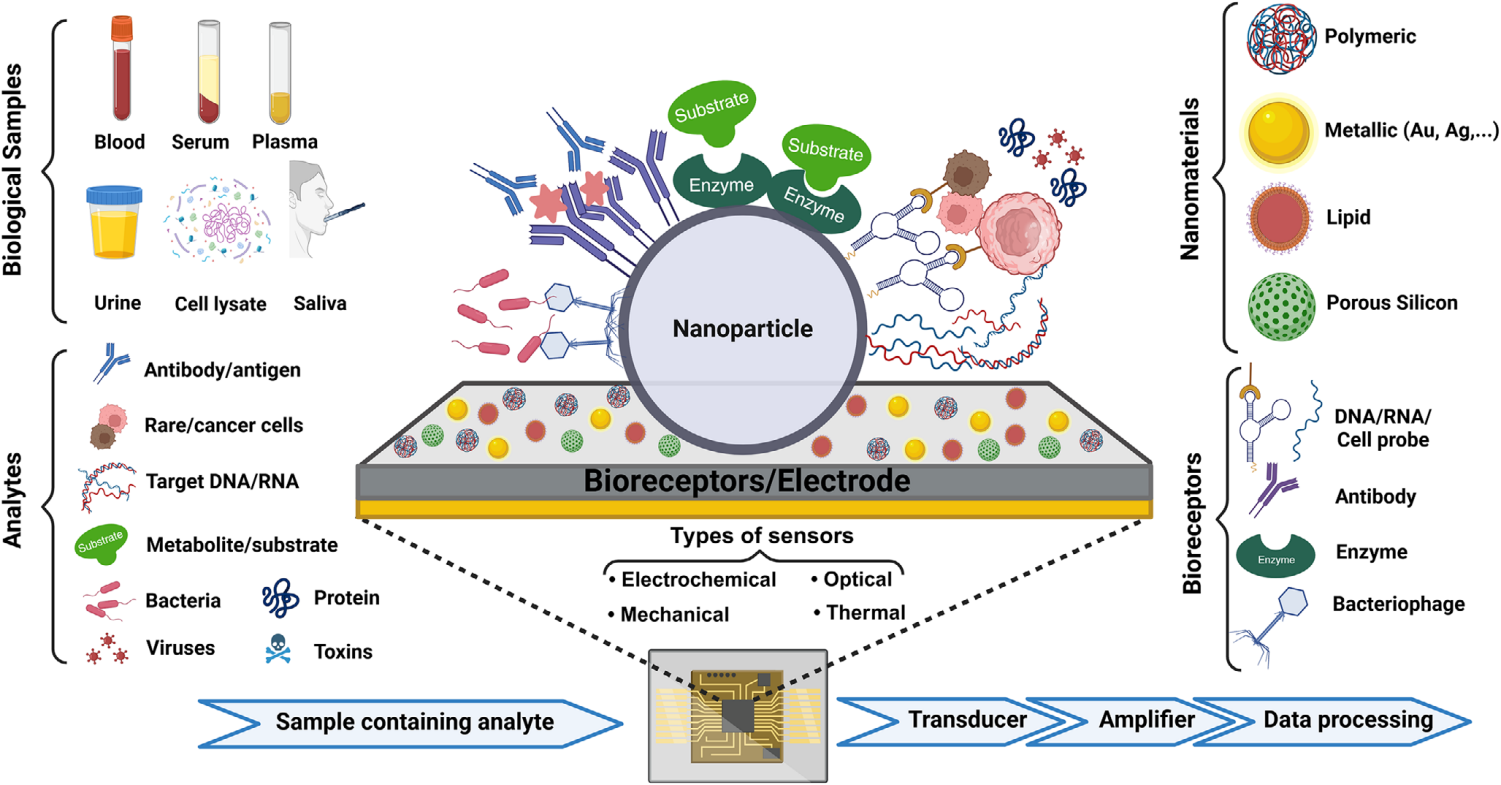
Schematic of the structure of a nanobiosensor and the process of biosensing involving common bioreceptors, samples, target analytes, and NPs. Figure created with BioRender.com.

Traditional biosensors cannot meet the demands required for these sensing applications, where very low detection limits are required against a high dynamic range of analytes due to the concentration‐limited diffusion flux of the targeted biomolecule toward a surface.^[^ ^12^ ^]^ A promising strategy to overcome the diffusion limit in the detection of single or very low‐concentration molecules is the development of nanobiosensors^[^ ^13^ ^]^ (**Figure** **1**). This class of biosensor utilize NPs that possess a high surface area‐to‐volume ratio, resulting in a larger sensing surface to biological fluid ratio than traditional planar sensor surfaces, thereby increasing the rate of analyte interactions and enabling the detection of ultra‐low concentrations (<pM) of target biomarkers.^[^ ^10^, ^15^ ^]^ For example, Morel et al., achieved approximately double the amount of protein A on a planar gold surface functionalized with gold nanoparticles compared to the gold planar surface alone.^[^ ^28^ ^]^ A useful area of future research would be more systematic comparisons between biosensors and nanobiosensors with the same sensor design, materials, target analyte, and other experimental conditions. The increase of the available sensing surface‐to‐volume ratio of nanosensors also enables them to be engineered into smaller end products than traditional sensors with similar sensing capabilities. This miniaturization can open up opportunities, particularly in wearable sensing, where small size can be an important factor.^[^ ^29^ ^]^ Similarly, the increased surface area to volume ratio of nanoparticles enables nanosensors to be designed that require less sample volume, which is important for conditions that require regular monitoring and for some clinically relevant fluids that are difficult to extract or in low abundance, such as spinal, cervico‐vaginal, nasal, and intraocular fluids.^[^ ^30^ ^]^ Small size is also an important consideration for designing devices with multiple components, such as when combining sensors with in‐built electronics or designing a multiplex sensing product capable of sensing different biomarkers simultaneously.^[^ ^2^, ^31^ ^]^

Beyond size‐specific benefits, nanobiosensors can also be designed to be dispersed in solution, effectively shortening the diffusion path and increasing the probability of encounter with the target analyte.^[^ ^13^ ^]^ This increases the rate of interactions and enhances detection and response time compared to conventional sensors that rely on analyte diffusion toward large planar surfaces.^[^ ^32^ ^]^ Because the NPs in dispersible biosensors need to be detached from other components, they either rely on functionalized NPs that can operate as both the biological recognition element and the transducing element (*e.g*., fluorescent or plasmonic NPs)^[^ ^1^, ^12^ ^]^ or utilize a mechanism where they can be collected post exposure, typically by using functionalized magnetic NPs and magnetic collection.^[^ ^13^ ^]^ For example, an electrically reconfigurable network of gold‐coated magnetic NPs was developed to enable ultrasensitive detection of microRNA and magnetic retrieval, with a detection limit as low as 10 am in whole blood.^[^ ^33^ ^]^ Evidently, nanobiosensors hold significant potential for clinical applications in disease diagnosis and management; however, for their successful translation into clinical settings, several challenges will need to be overcome.^[^ ^3^ ^]^ This has been delayed, in part, by the interference of the protein corona, which reduces their sensitivity and selectivity.

## 3 Formation of a Protein Corona on Nanobiosensors

**Figure 2:**
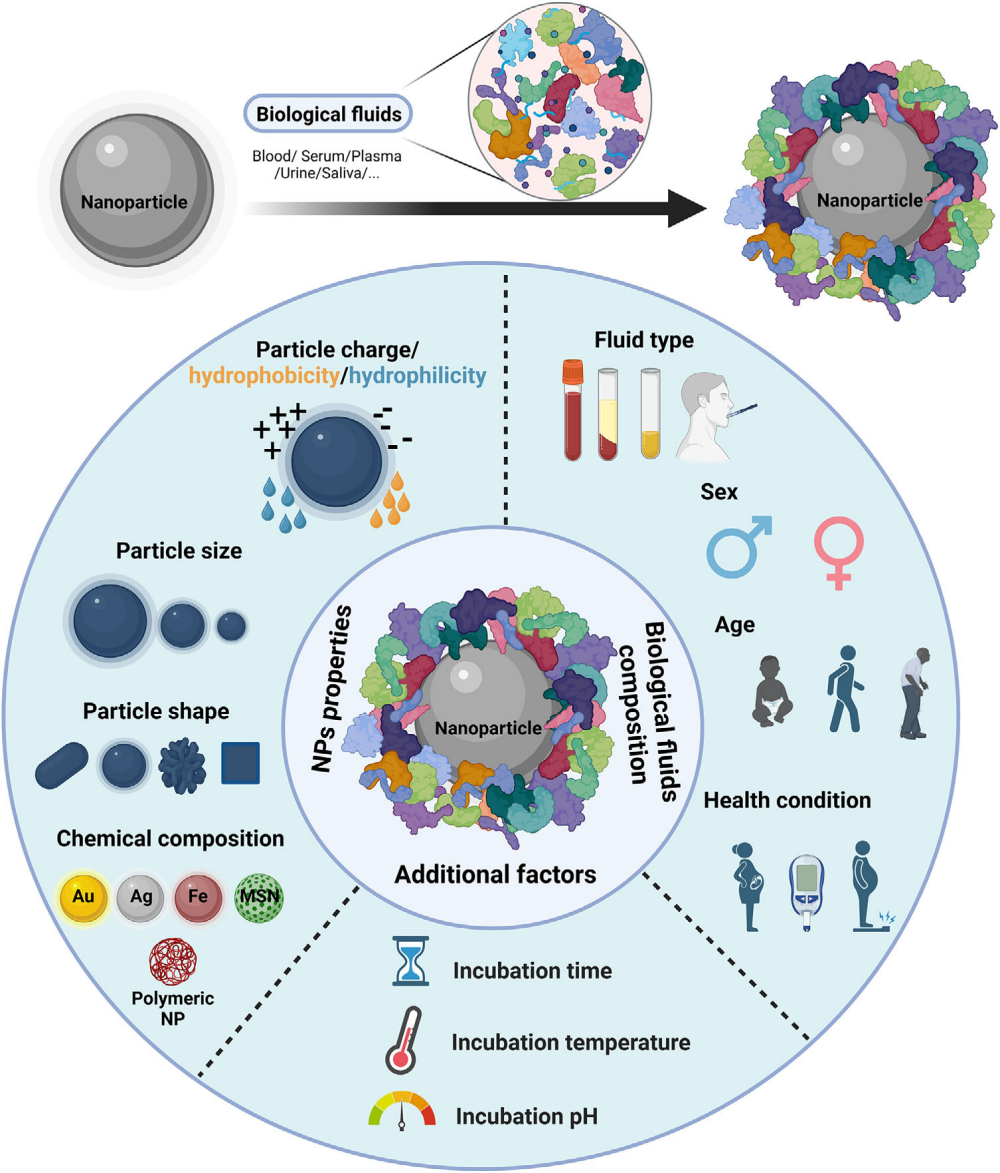
Schematic summary of the mechanism of the formation of a protein corona on NPs and key factors that influence corona formation. The three key factors include NPs’ physicochemical properties, biological fluids, and reaction conditions. Figure created with BioRender.com.

The protein corona forms and evolves in solution *via* a dynamic process influenced by the binding affinities and equilibrium constants of different types of proteins to the surface of NPs.^[^ ^5^, ^6^, ^34^ ^]^ This dynamic surface layer, which forms rapidly from highly mobile or abundant proteins, is termed the soft corona. This initial layer can be replaced over time by proteins that bind with comparatively higher affinity, constituting the hard corona.^[^ ^6^ ^]^ The physicochemical properties of the NPs, composition of the biological fluid, and additional factors such as time and exposure sequence play a role in shaping the composition of the corona,^[^ ^6^ ^]^ as summarized in **Figure** **2**.

Physicochemical properties include the nanomaterial shape,^[^ ^35^ ^]^ size,^[^ ^36^ ^]^ charge,^[^ ^37^ ^]^ the presence of functional groups,^[^ ^38^ ^]^ and composition (e.g., metal, polymeric, and lipid).^[^ ^39^ ^]^ These properties, which are interdependent^[^ ^40^ ^]^ can influence the thickness, composition, and decoration (e.g., protein conformation) of the corona. NP shapes with increased surface area‐to‐volume ratios demonstrate increased protein attachment compared to shapes with lower surface areas.^[^ ^35^ ^]^ However, shape curvature is also important to consider, with lower protein attachment detected on highly curved regions of NPs, such as sharp asperities^[^ ^41^ ^]^ due to steric hindrance, whereas there is preferential protein attachment on less curved surfaces.^[^ ^42^ ^]^ This demonstrates that protein attachment density can be heterogeneous around an NP. Interestingly, though smaller NPs have higher relative surface area, their increased curvature can result in fewer protein layers.^[^ ^42^, ^43^ ^]^ Surface functionalization also impacts corona formation. For example, Saha et al. reported that gold NPs pre‐coated with molecules possessing a linear organic end‐group adsorbed more protein than molecules with branched or cyclic end‐groups.^[^ ^38^ ^]^ NPs with more hydrophobic surfaces tend to adsorb more proteins, due to hydrophobic interactions.^[^ ^43^, ^44^ ^]^ However, this is not always the case as other forces such as electrostatic and H‐bonding are often more influential.^[^ ^45^, ^46^ ^]^ In comparison, hydrophilic NP surfaces can create a hydration layer surrounding the particles, which resists protein attachment.^[^ ^47^ ^]^ These layers are often composed of hydrated polymer brushes or other hydrated structures. Zwitterionic species, which possess negative and positive charges, create strongly hydrated surfaces due to electrostatic attractions with water molecules and therefore tend to hinder protein adsorption onto NPs,^[^ ^48^ ^]^ though exceptions do exist in certain ionic conditions. A strong surface charge (high or low) attracts more proteins, resulting in increased protein attachment, whereas a neutral surface charge tends to attract fewer proteins.^[^ ^46^ ^]^ This is partly due to these interactions typically taking place in high ionic strength solutions, which can screen electrostatic repulsion charges. Further details on the effect of NPs’ physicochemical properties on protein corona formation are provided by Bilardo et al.^[^ ^46^ ^]^ In addition to corona thickness, corona composition varies according to the physicochemical properties of NPs. For example, Lundqvist et al. found that NPs with different sizes and surface charges had key differences in the protein corona composition.^[^ ^49^ ^]^ There are various examples where NP size,^[^ ^50^ ^]^ hydrophobicity,^[^ ^43^ ^]^ and shape^[^ ^51^ ^]^ influence the protein corona composition.

Besides the physicochemical properties of NPs, biological conditions can influence the protein corona thickness, composition, and decoration. For example, different compositions of corona are formed even when identical NPs are exposed to different biofluids such as blood,^[^ ^52^, ^53^ ^]^ lymph,^[^ ^52^ ^]^ serum,^[^ ^53^, ^54^ ^]^ and plasma.^[^ ^53^, ^54^ ^]^ For instance, Bonvin et al. analyzed the formation of a protein corona on iron oxide NPs exposed to blood and lymph. The authors found that biofluid composition significantly influenced protein corona composition.^[^ ^52^ ^]^ They also observed differences in corona composition between NPs exposed to the same biofluids but under different flow rates. The authors proposed a protein force‐promoting binding mechanism that is dependent on flow. The influence of flow rate is an important consideration when designing nanobiosensors to minimize nonspecific protein binding when working in a biological milieu, particularly as flow is often a controllable parameter in a biosensing system. Biofluid concentration can also influence corona formation and composition.^[^ ^55^ ^]^ The source of the biofluid is also important, as protein corona composition from the same type of fluid derived from different individuals can differ.^[^ ^6^ ^]^ Interestingly, this has developed the idea of a “personalized” or “disease‐specific” protein corona.^[^ ^56^ ^]^ This concept will be discussed in more detail in the section below: *Exploiting the protein corona for biosensing*. Further, the source species is also important, with protein coronas formed on nanomaterials in animals, plants, and the natural environment displaying notable differences.^[^ ^57^ ^]^ This has contributed to the terminology of the “eco‐corona” being developed.^[^ ^58^ ^]^ Other conditions, such as temperature,^[^ ^59^ ^]^ pH,^[^ ^60^ ^]^ time,^[^ ^34^, ^55^, ^61^ ^]^ and NP journey,^[^ ^52^ ^]^ can have a substantial influence on the protein corona formation and evolution. For example Oberländer et al. reported differences in the composition of coronas formed in media at 4 and 37 °C, with the former corona having a higher proportion of coagulation proteins.^[^ ^62^ ^]^ The authors proposed that the binding affinity of different proteins changed with the different temperatures due to individual protein conformational changes.^[^ ^62^ ^]^ The pH of a solution can alter the surface charge of NPs and proteins, as well as potentially causing proteins to denature, which can influence the corona composition and binding kinetics.^[^ ^60^ ^]^ These studies demonstrate that NPs, physicochemical properties, biofluid composition, and incubation conditions are all important considerations for corona formation on nanobiosensors. Further studies are necessary to better understand how these conditions influence protein corona formation and consequently, the performance of nanobiosensors. This understanding could enable the rational design of nanobiosensors that either minimize or exploit the effect of protein corona formation. For example, trialing different flow rates, exposure times, temperatures, pre‐processing of biofluids, and the addition of washing steps may influence the performance of biosensors.

## 4 Nanobiosensor Challenges Arising from Corona Formation

As discussed previously, nanobiosensors are a useful tool for detecting low levels of biomarkers in complex solutions. However, the formation of a protein corona when analyzing biological fluids can reduce the performance of the sensor, including their lower detection limit.^[^ ^63^, ^64^ ^]^ The main reason for the reduced performance is that the protein corona can adversely influence the binding kinetics between target analytes and bioreceptors on the NPs’ surface.^[^ ^33^, ^65^ ^]^ For example, Tao et al. found a loss of up to 80% in the detection signal when using gold NPs in buffer solutions containing representative highly abundant plasma proteins (*e.g*., bovine serum albumin, fibrinogen, hemoglobin, and β‐lactoglobulin) compared to protein‐free solutions.^[^ ^63^ ^]^ A similar study using gold‐coated magnetic NPs demonstrated a reduction in specific targeting of the programmed death‐ligand 1 in whole blood compared to a simple buffer (PBS) media.^[^ ^66^ ^]^ In these cases, the receptor is blocked from interacting with the analyte due to non‐specific binding and molecular crowding effects.^[^ ^67^ ^]^ There have been several models developed to help understand and predict binding kinetics of a system, however it remains a difficult task due to the many permutations in conditions, such as analyte and background media concentration, temperature, flow rate, molecular degrees of freedom, and chemical potential, among many others, as reviewed therein.^[^ ^67^ ^]^

**Figure 3:**
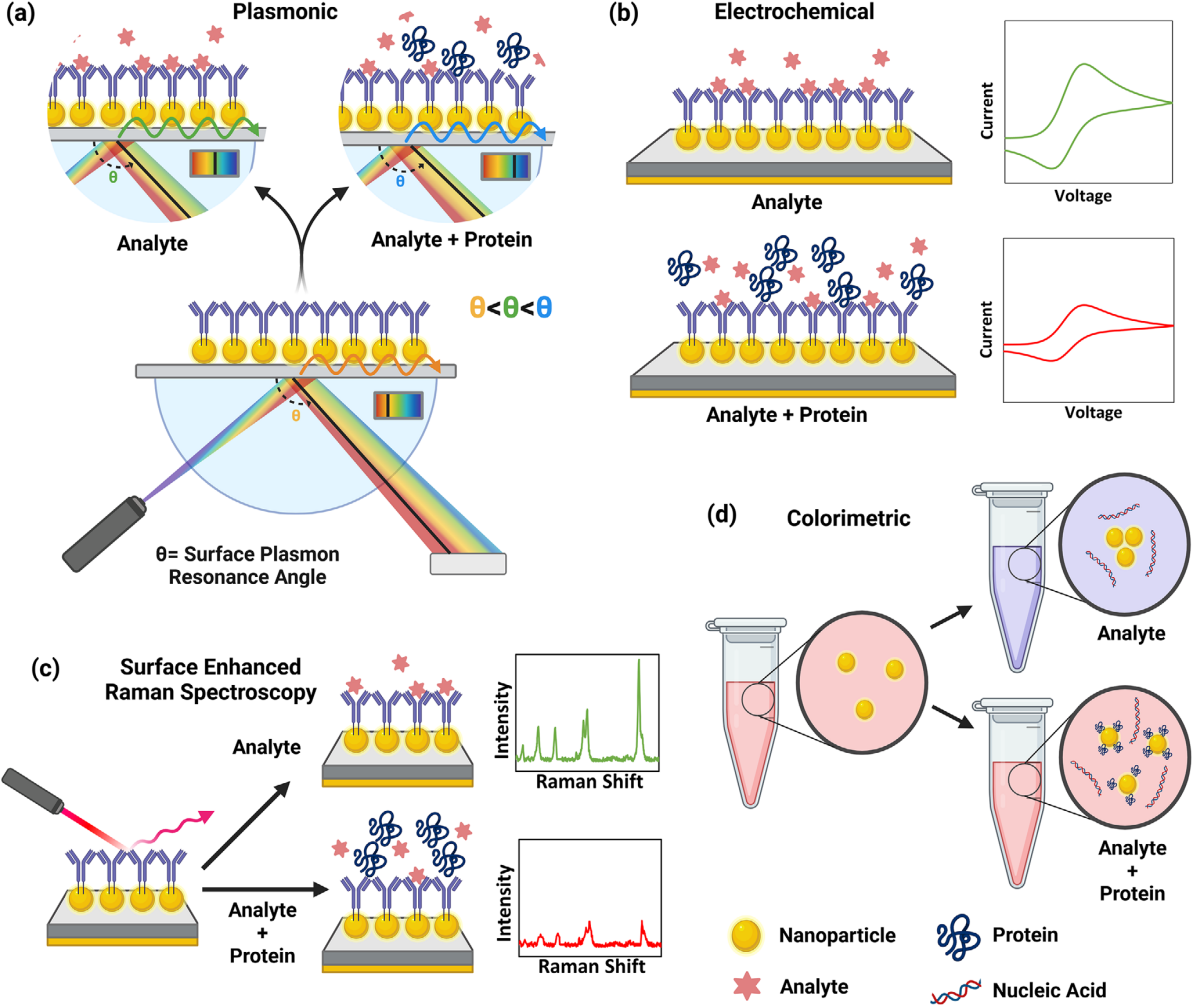
Demonstration of the influence of the protein corona on nanobiosensors, including a) plasmonic, b) electrochemical, c) SERS, and d) colorimetric sensors. In this illustration, various sensors’ outputs are adversely impacted by the formation of protein corona (for example, the absence of a signal as opposed to a reduced or altered signal). Figure created with BioRender.com.

In addition to blocking receptor–analyte interactions, the protein corona can impact nanobiosensors in other ways such as influencing the surface properties of NPs, which are often directly related to signal generation^[^ ^68^ ^]^ (**Figure** **3**). For example, NP‐based electrochemical biosensors rely on charge transfer through the NP surface, and the nonspecific binding of the protein corona can interfere with this charge transfer.^[^ ^69^ ^]^ Similarly, nanobiosensors that use surface‐enhanced Raman spectroscopy (SERS) rely on the NP surface.^[^ ^69^ ^]^ Importantly, the reduced performance of the sensors can result in either no signal or a change in signal. For example, Pancaro et al. found that a localized surface plasmon resonance (LSPR) nanobiosensor based on gold nanorods displayed either no signal or a blue‐shift in human serum compared to a serum‐free buffer (10 mm HEPES).^[^ ^70^ ^]^ This propensity for different outputs following corona formation makes it difficult to predict the exact effect of the protein corona on nanobiosensor outputs. The unfavorable effect of protein corona formation has been most widely reported in colorimetric biosensors^[^ ^71^, ^72^ ^]^ due to masking effects on the NP surface. For example, Wang et al. found that their label‐free gold NP‐based colorimetric biosensor was effective for the detection of DNA in simple buffer but ineffective in serum.^[^ ^71^ ^]^ The NP sensor relied on selective aggregation in the presence of the target DNA; however, in the complex biological media, the proteins would coat the NPs, providing colloidal stability and preventing colorimetric color change. Overall, impaired NP surface properties due to the protein corona impact the interpretation of results and can reduce biosensor sensitivity, selectivity, and overall accuracy and reliability.

Beyond human health, protein corona formation is also an issue in nanobiosensors for plants.^[^ ^57^ ^]^ In many cases, these sensors are placed *in planta* and are useful for continuous monitoring of plant stress biomarkers,^[^ ^73^ ^]^ analytes,^[^ ^74^ ^]^ pests,^[^ ^75^ ^]^ pathogens,^[^ ^76^ ^]^ and other markers in the plant to indicate plant health. *In planta* sensors can also measure important properties of soil, such as toxins.^[^ ^77^, ^78^ ^]^ For example, Wong et al. were able to detect nitroaromatics, which are a concerning contaminant in soil and groundwater, and accumulate in the mesophyll.^[^ ^77^ ^]^ Notably, they developed a sensor through corona phase molecular recognition, by forming a corona of the Bombolitin II peptide around single‐walled carbon nanotubes, prior to introduction into the plant, that functioned as a near‐infrared fluorescent nanosensor for nitroaromatics recognition.^[^ ^77^ ^]^ Plants have different transport pathways and translocation barriers, meaning that the impact of the protein corona will differ *in planta* to in vivo for disperesed NPs. For sensors, the biological environment, such as the composition of the plant millieu, pH, temperature, flow, and ionic strength, can be quite different from human biofluids.^[^ ^57^ ^]^ These differences have, in part, contributed to the terminology of the “eco‐corona” being developed, which describes the protein corona formed on nanomaterials in environmental contexts.^[^ ^58^ ^]^ Understanding the effect of the eco‐corona on plant biosensors and how this can be potentially exploited would be an interesting and useful direction for future research.^[^ ^6^, ^14^, ^57^ ^]^

## 5 Understanding Protein Corona Formation on Nanobiosensors

High quality and reproducible analyses of protein corona formation, composition, and other properties are important for comparison between studies. There are many relevant techniques suitable for characterizing the protein corona, as briefly summarized in **Table** **1**. These techniques include the visualization of corona formation, such as *via* microscopy, understanding of physicochemical properties of the NP–protein corona complexes, and specific information about the protein composition, such as *via* mass spectrometry.^[^ ^79^ ^]^ A detailed discussion of protein corona characterization is provided therein.^[^ ^6^, ^79^, ^80^ ^]^ In addition to the conventional techniques, several nanobiosensing strategies have been developed recently specifically for analyzing protein corona formation.^[^ ^81^, ^82^, ^83^, ^84^ ^]^ These strategies involve LSPR spectroscopy,^[^ ^81^ ^]^ SERS,^[^ ^82^ ^]^ NP‐enhanced laser‐induced breakdown spectroscopy (NELIBS)^[^ ^83^ ^]^ and custom biosensors and workflows.^[^ ^84^ ^]^ Moving forward, the nanobiosensing field has the potential to contribute further to the understanding of protein corona formation (for nanobiosensing and other research).

**Table 1:** Summary of techniques to analyze the protein corona

Technique	Principle	Information for protein corona	Refs.
Transmission electron microscopy (TEM)	Passes high‐energy electrons through a sample and detects electron scattering to generate ultrahigh resolution images	Structure visualization Sub‐nanometer resolution	[85]
Cryo‐TEM	Rapidly freezes and sublimates samples prior to TEM imaging to enable sample imaging closer to the native liquid state	Structure visualization in close to native conditions Sub‐nanometer resolution	[86]
Atomic force microscopy (AFM)	Uses a nanoscale tip to scan a surface to generate high‐resolution 3D surface scans, mechanical, and interaction data	High‐resolution 3D scans Sub‐nanometer resolution Nanomechanical information Interaction information (*e.g*., adhesion)	[87, 88]
Dynamic light scattering (DLS)	Converts Brownian motion of particles into different solutions to estimate particle size	Particle size characterization	[34, 36, 89]
Fourier transform infrared (FTIR) spectroscopy	Detects the absorbance or transmittance of light in the infrared spectrum when passed through a sample, indicating specific molecular bonds	Peak intensity and wavelength indicate chemical information about bound proteins	[90]
Ultraviolet visible (UV–Vis) light spectroscopy	Measures absorbance/transmission of light in the UV and visible spectra when passed through a sample, indicating concentration or molecular identity	Colloidal stability analysis Indication of bound proteins from peak intensity and wavelength	[91]
Surface enhanced Raman spectroscopy (SERS)	Detects changes in light wavelengths emitted from nanostructured surfaces, providing sensitive information on molecule–NP interactions	Binding information Understanding of protein secondary structure	[92]
Liquid chromatography‐mass spectrometry (LC‐MS/MS)	Distinguishes between different molecules owing to differences of the mass/charge (*m*/*z*) ratio	Identify and quantify proteins and peptides (for bottom‐up proteomics method) Identify and quantify proteoforms (for top‐down proteomics method) Main technique for analyzing the proteomic composition of the protein corona	[93, 94]
Gel electrophoresis	Separates biological molecules according to their size	Information on the types of proteins attached (based on size)	[95]
Quartz crystal microbalance (QCM)	Utilizes a quartz crystal that resonates at high frequency to sensitively detect mass changes on a surface, which changes the frequency	Detection of very small mass changes as proteins are adsorbed onto particles Useful for corona formation kinetics	[96]
Zeta potential measurement	Measures the potential at the slipping plane of particles	Detection of changes in charge on the surface of NPs Possible indication of particle stability	[88]
Thermogravimetric analysis (TGA)	Records changes in weight of a sample with respect to temperature or time, owing to loss of gas, water, or breakdown of the material	Possible determination of mass increases of NPs owing to protein adsorption	[97]
Isothermal titration calorimetry (ITC)	Quantifies intermolecular interactions by measuring heat released or absorbed during the titration of one reactant into another reactant under constant temperature and pressure conditions	Possible binding information between proteins and NPs, including binding affinity and interaction mechanisms	[43, 98]
Differential scanning calorimetry (DSC)	Quantifies the heat capacity of a sample based on the difference in energy required to equalize the temperature between the sample and reference material	Conformational information of adsorbed proteins by determining their unfolding temperature	[99]
X‐ray photoelectron spectroscopy (XPS)	Samples are bombarded with high‐energy X‐rays, causing electron emission with specific energy relative to their chemical composition	Useful for understanding surface chemistry	[100]

Besides experimental techniques, machine learning and artificial intelligence (AI) have recently emerged as a useful tool to understand and predict the composition of the protein corona under different conditions.^[^ ^101^ ^]^ For instance, Ban et al. presented a machine learning workflow, utilizing the random forest method to predict corona composition, with a focus on NPs with and without functionalized surfaces.^[^ ^102^ ^]^ Other researchers have also used the random forest approach,^[^ ^103^ ^]^ as well as other approaches.^[^ ^104^ ^]^ The key takeaway from the current literature is that there is substantial work remaining to develop functionally useful predictive models for corona composition. This will become increasingly important in many areas but specifically in biosensing where it can be used to assess the effectiveness of potential materials and designs of corona formation. Further, predictive models could be a useful tool to design multiplexing nanobiosensors for detection and measurement of the protein corona components. There is also a challenge with predicting protein corona composition as proteins are only metastable and can unfold and refold into different secondary and tertiary structures to become more thermodynamically stable.^[^ ^105^ ^]^ This changes the exposed residues and surface properties of the protein and therefore how it interacts with NPs.^[^ ^106^ ^]^ In the future, programs such as AlphaFold^[^ ^107^ ^]^ could be leveraged to understand protein shape and folding under varied conditions to obtain a greater understanding of steric hindrance and charged/hydrophobic residues in proteins with different structures.

Despite the many tools available for analysis, in the nanomedicine field, there is a lack of standardization between studies and inherent issues with certain methodologies and interpretations of the results that can lead to misleading or incomparable results, as highlighted in a recent comment piece.^[^ ^108^ ^]^ Recent studies have tried to establish standardized protocols to characterize the protein corona,^[^ ^79^, ^108^ ^]^ and more specifically within the fields of nanomedicine and drug delivery.^[^ ^108^, ^109^ ^]^ Although these protocols are useful, within the field of nanomedicine, analysis for nanobiosensing applications can be different, and specific protocols are lacking. Moreover, basic analysis of the protein corona in biosensing applications is rare and thus signifies an important gap in the field of nanoscale biosensing. In addition to standardization, it is essential to incorporate specific control samples and experiments to prevent protein contamination from interfering with the sensing process, as such contamination can lead to inaccuracies in sensor signals.^[^ ^110^ ^]^

## 6 Minimizing the Adverse Effects of Protein Corona Formation on Nanobiosensors

Preventing protein corona formation is a promising strategy to ensure the function of nanobiosensors in a complex biological milieu. Many of these strategies are extensions of previous efforts to prevent biofouling of surfaces and typically involve changing the surface chemistry of NPs,^[^ ^38^, ^91^, ^111^, ^112^ ^]^ the addition of stealth polymers,^[^ ^113^ ^]^ focusing on zwitterionic polymers,^[^ ^111^, ^114^ ^]^ protein pre‐coating strategies,^[^ ^115^ ^]^ and physical modifications.^[^ ^55^ ^]^ Although these strategies can be promising for nanomedicine, in biosensing applications, the added coating layers can alter the surface properties of the NPs, such as reducing the electrochemical or plasmonic sensitivity or negatively influencing the sensing performance.^[^ ^116^ ^]^ For example, the poor conductivity of polymer coatings on electrochemical sensors, owing to the insulating polymer backbones, can slow electron transfer kinetics and reduce electrochemical signals.^[^ ^117^ ^]^

Strategies to reduce the adverse effects of protein corona formation on nanobiosensors can differ from other bio‐applications of NPs, particularly because the NPs can be washed or further processed. This contrasts with NPs that are used for drug delivery, where prevention or control of the protein corona is the only viable strategy, as removal of the adsorbed proteins is quite challenging once the NPs are administered into the body. However, in the case of nanobiosensors, the NPs that interact with the biological fluid can then be washed, or in the case of dispersed nanobiosensors, recollected and further processed. This offers the opportunity for a corona removal step.^[^ ^118^ ^]^ Currently, there are no established strategies or protocols for the removal or partial removal of the protein corona in the nanobiosensing literature. The field could benefit from studies that systematically evaluate the effectiveness of different washing protocols for NPs specifically targeted toward biosensing applications.

## 7 Exploiting the Protein Corona for Biosensing

**Figure 4:**
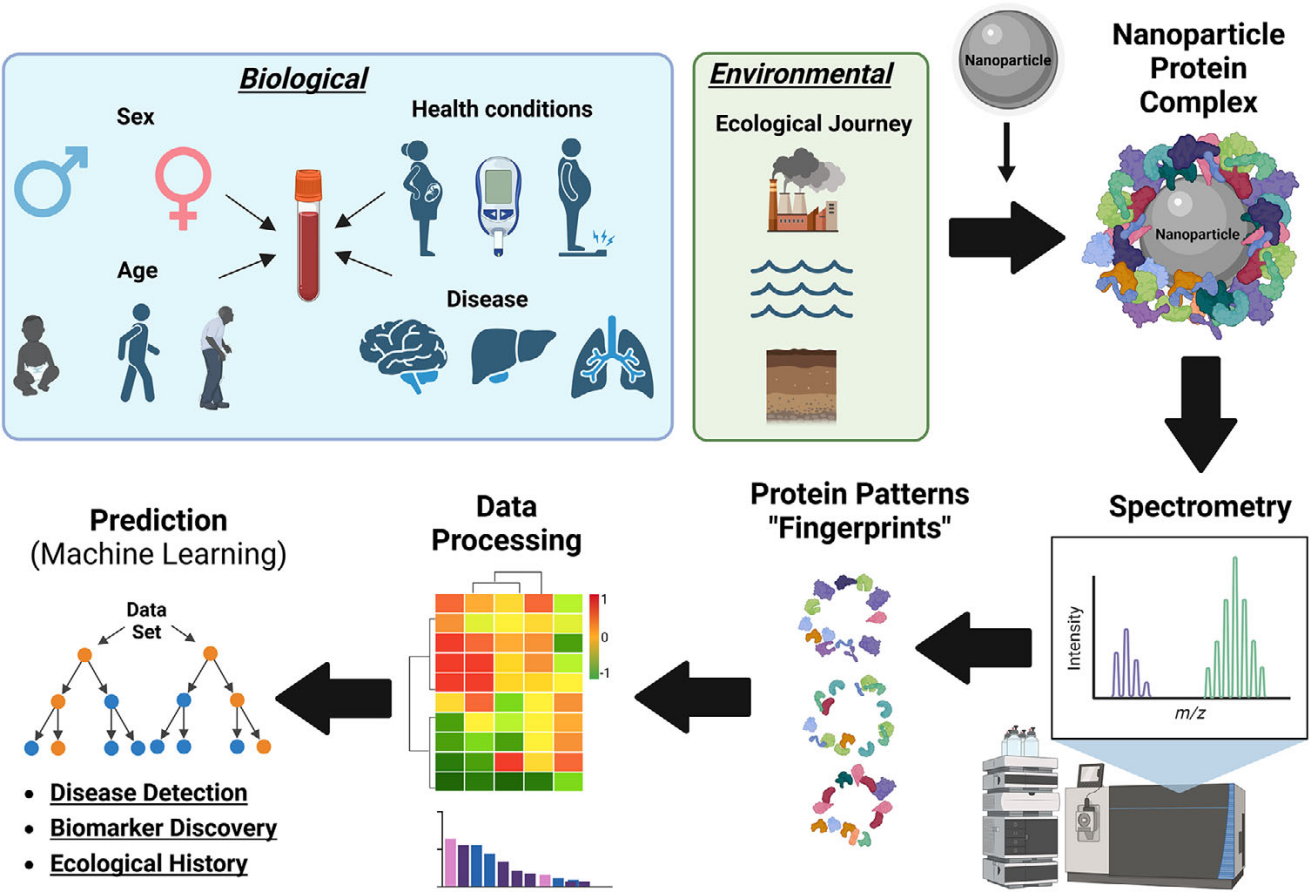
Schematic summary of the use of a “personalized” protein corona for biosensing. When NPs are exposed to different fluids from different biological sources or environmental settings, a protein corona forms that is specific to the sex, age, and health status of an individual, or ecological journey. The protein corona can be processed through mass spectrometry, creating unique protein patterns or “fingerprints” that can be processed and used for disease detection, biomarker discovery, and the understanding of the ecological journey of an NP. Figure inspired by refs. [6, 119, 137]. Figure created with BioRender.com.

As described earlier, the type of biological fluid is an important factor for protein corona formation and composition. Studies have also shown that the same biofluid derived from different sources can result in distinct protein corona compositions on the surface of identical NPs, reflecting the different protein profiles between individuals. This has led to the concept of the “personalized corona” first introduced by Hajipour et al. in 2014.^[^ ^56^ ^]^ That is, the composition of the protein corona formed on NPs when exposed to a biological fluid is specific to the individual and can be dictated by factors such as sex, age, and health status. Interestingly, the proportion of rare biomarkers compared to highly abundant proteins such as albumin can be substantially increased in these protein coronas from the original biofluid through nanomaterial selection and sample processing, meaning that differences between samples can be more easily identified.^[^ ^119^ ^]^ Several subsequent studies have investigated the clinical implications of a personalized corona, including investigating differences in NP protein coronas formed from plasma derived from donors with and without specific diseases such as cancer^[^ ^120^ ^]^ as reviewed therein.^[^ ^121^ ^]^ Notably, this concept has been exploited recently to develop multi‐array disease nanobiosensors.^[^ ^95^, ^122^, ^123^, ^124^, ^125^ ^]^ This concept was first demonstrated in 2019, where Caracciolo et al. investigated protein corona formation on NPs that were exposed to plasma from individuals with different cancers and healthy donors and demonstrated the early disease detection capability of the nanobiosensors using the personalized coronas.^[^ ^122^ ^]^ Specifically, three liposomes with different charges (cationic, anionic, neutral) were designed, and the identity of proteins that attached to the liposomes upon their exposure in plasma samples from different individuals was determined though mass spectrometry‐based proteomics. The data were subjected to statistical analysis, including partial least squares discriminant analysis (PLS‐DA) as a linear projection method, which is a useful tool for omics data to reduce the data dimensionality,^[^ ^126^ ^]^ in this case reducing the proteomics data to the top 69 most important variables. Proteomics data from 30 training samples were then analyzed by PLS‐DA using this 69‐D variable space and were successfully assigned to six classes (cancers) with >99% classification accuracy. This method was further verified through the use of counter‐propagation artificial neural network (CPANN) analysis, which is a non‐linear mapping process that enables visualization of high‐dimensional data in a 2D neural grid,^[^ ^127^ ^]^ which aligned with the PLS‐DA results in discriminating six cancers using the selected 69 variables. Both the PLS‐DA and CPANN were then applied to proteomics data from 15 test samples and were able to classify them into brain, lung and pancreas cancer groups. Collectively, the modelling informed that the protein corona NP multi‐sensor array could develop specific protein profiles or “fingerprints”, which could discriminate between samples of different cancers and those of healthy donors. The disease fingerprinting concept that was first introduced in that study has been further developed by other researchers for the detection of different cancers including breast,^[^ ^123^ ^]^ prostate,^[^ ^123^ ^]^ lung,^[^ ^128^ ^]^ pancreatic,^[^ ^124^ ^]^ and brain,^[^ ^95^ ^]^ as well as other diseases such as sepsis.^[^ ^129^ ^]^ It is summarized in **Figure** **4**.

While the protein corona fingerprint method is the prime candidate for exploiting the corona for biosensing applications, there are other approaches to developing sensors that utilize the forming of a protein corona on nanomaterials. For example, Shuai et al. used protein corona formation as part of the synthesis method of upconversion NPs made from a combination of Yb, Gd, Y, and Er, with a protein corona comprised of silk fibroin for aptamer‐free detection of antibiotic residues.^[^ ^130^ ^]^ Further, Zhang et al. were able to detect metallothionens, which are a group of cysteine‐rich proteins that are useful biomarkers for heavy metal poisoning, through a colorimetric sensor sensitive to metallothionen protein corona formation utilizing polymer‐caged gold NPs.^[^ ^131^ ^]^ Quagliarini et al., demonstrated cancer detection through the measurement of changes in magnetic levitation of nanomaterials due to differing protein corona formation.^[^ ^132^ ^]^ The concept of utilizing the protein corona as a key component of a sensor has also expanded beyond biomedical applications. For example, Xiao et al. designed a proof‐of‐concept photoelectrochemical sensor to detect microplastic pollution of aquatic environments based on protein corona‐like interactions between polystyrene particles (50–200 nm in diameter) and bovine serum albumin.^[^ ^125^ ^]^ The use of the protein corona for sensing has also been applied to design an impedance biosensor for the detection of Gram‐positive bacteria^[^ ^133^ ^]^ and a nanoplasmonic paper‐based biosensor for the detection of nonspecific biomacromolecules with a targeted application as an inexpensive system for cleanliness evaluation for hospitality, healthcare, and other industries.^[^ ^134^ ^]^ These methods are not as robust as the fingerprint method, however may have utility as low‐cost, simple sensors.

In addition to disease diagnosis, the use of the protein corona to sub‐sample the proteome can enable amplification of protein biomarkers that exist at very low concentrations, typically in the plasma.^[^ ^18^, ^94^, ^119^, ^135^, ^136^ ^]^ For example, Blume et al. utilized three different super paramagnetic iron oxide NPs (SPIONS) functionalized with tetraethyl orthosilicate, poly(dimethylaminopropyl methacrylamide) or poly(ethylene glycol) to generate negative, positive, and neutral surface chemistries, respectively, for deep proteomic profiling, identifying differences between samples derived from healthy donors and donors with early non‐small‐cell lung cancer.^[^ ^119^ ^]^ Typically, these biomarkers are present at too low concentrations that cannot be detected using traditional proteomics approaches, and the exploitation of protein corona formation on NPs has enabled the suggestion of new potential biomarkers for different diseases such as Alzheimer's,^[^ ^137^ ^]^ multiple system atrophy,^[^ ^138^ ^]^ and a range of cancers.^[^ ^135^, ^139^ ^]^

Exploiting the protein corona for use as a sensor, particularly through the multi‐array fingerprinting approach, has clear potential; however there are important challenges that need to be overcome before these kinds of sensors can be used clinically. A major challenge is ensuring reproducibility and accuracy given the natural broad variability of protein profiles between individuals due to multiple factors beyond the target condition such as age, diet, gender, and other health factors. This issue may be mitigated by using a large number of diverse patient samples to train on, to establish reliable protein fingerprints for specific conditions.^[^ ^122^ ^]^ However, such datasets are not always available, such as for rare conditions or where biobanks do not contain samples from diverse populations (age, gender, ethnicity, health status, etc),^[^ ^140^, ^141^ ^]^ though there is a coordinated effort to improve biobank diversity.^[^ ^141^ ^]^ Another issue is variability between proteomic facilities as demonstrated in a study by Ashkarran et al. where they sent identical protein corona samples to 17 different proteomic core facilities across the USA and found only 1.8% of the total proteins identified (73/4022) were shared across all 17 facilities.^[^ ^93^ ^]^ To address this, the same group has developed a harmonized data processing pipeline which increased detection to 7.1% of total proteins across 15 facilities,^[^ ^142^ ^]^ though there is still room for improvement.^[^ ^108^ ^]^ These multi‐array sensors also demonstrate a change in disease prediction compared to sensors that detect individual biomarkers, where direct relationships between the biomarker and the disease are easier to establish. In comparison, it is more difficult to establish a causal relationship between a protein fingerprint and the disease state from a mechanistic perspective. On the other hand, though more difficult to establish, these characteristic protein profiles of disease may be able to provide a deeper understanding of a certain disease from an omics level. Another similar challenge arises when considering a lot of these approaches utilize artificial intelligence for their data analysis. Such analysis can result in a lack of transparency in clinical decision making, making it harder to identify when and importantly why a model makes an error.^[^ ^143^ ^]^ Finally, a further fundamental understanding of these sensors is recommended such as investigating different diseases, biofluids, disease states, nanomaterial designs, work flows, data analysis, or even how the amount of protein attached may affect their performance. Challenges are always expected of new biomedical technologies and the issues facing protein corona sensor technologies may be overcome with targeted future research.^[^ ^19^, ^144^ ^]^

## 8 Conclusions and Future Perspectives

Nanobiosensors are a powerful tool in clinical settings for disease diagnosis and management. However, they develop a protein corona upon exposure to biomolecules present in biological fluids, which can reduce their sensitivity and selectivity. A comprehensive understanding of the effect of the protein corona on nanobiosensors is lacking, partly because of the infancy of the field of nanobiosensors and protein coronas, simplified biosensor experimental designs, and technical challenges of characterizing the protein corona. However, understanding this impact is essential to fulfill the potential of nanobiosensors and enable their use for medical diagnostics in complex biological media. To minimize the impact of corona formation on sensing applications, a better understanding is needed on protein corona formation and how the properties of nanomaterials and biological fluid impact this process. The design of NP systems with tailored surface properties to influence the composition and characteristics of the protein corona is key to its prevention or utilization for finger printing. By modulating the corona, there is an opportunity to enhance the performance and specificity of sensing platforms. Developing advanced computational and statistical approaches are emerging as an important tool to analyze the complex datasets generated from protein corona profiling and extract meaningful information. The generation of experimental data sets over the coming years is expected to lead to improved predictions from machine learning and AI models.

The biggest opportunity for nanobiosensing with regard to the protein corona is to take advantage of the protein corona formation process itself. The development of protein corona sensing utilizing personalized coronas has opened up opportunities for disease diagnosis, biomarker discovery, and analysis of NP ecological journeys. The fingerprinting type of sensing is projected to develop quickly with advances in AI‐based technologies and optimization of processes. However, these technologies need to address challenges such as the need for large training samples, variability across proteomics facilities, and other ethical and regulatory hurdles before their clinical adoption. Beyond the fingerprinting methodology, the detection of the protein corona for other sensing applications also shows promise. To further the understanding and exploitation of the protein corona in biosensing, we encourage the research community to establish standardized protocols for protein corona analysis toward advancing diagnostics, health monitoring, and personalized medicine in the future.

## Conflict of Interest

M.M. discloses that (1) he is a co‐founder and director of the Academic Parity Movement (www.paritymovement.org), a non‐profit organization dedicated to addressing academic bullying and harassment; (2) he is a co‐founder of and shareholder in Targets’ Tip and AlbuDerm; and (3) he receives royalties/honoraria for his published books, plenary lectures and licensed patents.

## Data Availability

The data that support the findings of this study are aailable from the corresponding author upon reasonable request.
